# Taxonomy and phylogeny of *Sanguinoderma rugosum* complex with descriptions of a new species and a new combination

**DOI:** 10.3389/fmicb.2022.1087212

**Published:** 2022-12-21

**Authors:** Yi-Fei Sun, Yu-Xuan Fang, Bao-Kai Cui

**Affiliations:** Institute of Microbiology, School of Ecology and Nature Conservation, Beijing Forestry University, Beijing, China

**Keywords:** Ganodermataceae, macrofungi, morphology, multi-gene phylogeny, new taxa

## Abstract

*Sanguinoderma* is distributed in tropical and subtropical areas as a member of *Amauroderma* s. lat., and the economic values of *Sanguinoderma* led to high attention in the taxonomic studies. Previously, 16 species have been developed into *Sanguinoderma*. In this study, the taxonomic system of *Sanguinoderma* was reconducted based on morphological and multi-gene phylogenetic analyses, especially making a distinction for *Sanguinoderma rugosum* complex. Morphological analysis was based on the notes of macro- and micro morphological observations. Multi-gene phylogenetic analyses were used maximum likelihood (ML) and Bayesian inference (BI) analyses inferred from combined dataset of ITS, nLSU, *rpb2, tef1*, mtSSU, and nSSU. Combined with morphological characters and phylogenetic evidence, the results demonstrated that *S. rugosum* complex consists of five taxa, in which *Sanguinoderma leucomarginatum* was described as a new species, and it is characterized by the orbicular pilei with white to buff margin when fresh and clavate apical cells of pileipellis with septa. In addition, *Amauroderma preussii* was transferred to *Sanguinoderma* as a new combination due to its blood-red color-changed pore surface; it is characterized by the funnel-shaped, greyish brown, and glabrous pilei with strongly incurved margin. Detailed descriptions and photographs of the two species were provided. With the extension of this study, 18 species were accepted in *Sanguinoderma*, and 12 species among them were distributed in China. A key to accepted species of *Sanguinoderma* was also provided.

## Introduction

Ganodermataceae is an important family of macrofungi according to its high economic and ecological values. Some species in this family, such as *Ganoderma lingzhi, Ganoderma sinense, Ganoderma tsugae, Amauroderma rude*, and *Amauroderma rugosum*, have been domesticated successfully in China and commonly used as traditional medicine for anti-cancer treatment, for lowering blood pressure, and for improving immunity (Wang et al., 1993; Dai et al., 2009; Cao et al., 2012; Chan et al., 2013; Jiao et al., 2013; Li et al., 2015; Zhao et al., 2015; Fung et al., 2017; Xiao et al., 2017; Zhang et al., 2019). As white-rot fungi, some species like *G. australe, G. lingzhi, G. lucidum*, and *A. rugosum* can secrete a series of carbohydrate hydrolase, peroxidase enzymes, and laccases to degrade the organic matters in forests, and this performance has been widely used as biofuel, for industrial applications and pollution abatement (Jong et al., 2017; Si et al., 2019, 2021; Wang et al., 2021). Besides, *Ganoderma boninense, Ganoderma philippii*, and *A. rugosum* as pathogenic species in Ganodermataceae can cause stem rot or root rot in forests leading to economic damage (Pilotti, 2005; Glen et al., 2009; Abubakar et al., 2022). To further understand how the economic and ecological values produced by Ganodermataceae species, genomics, transcriptomics, and proteomics were introduced by biologists to explore the mechanism of evolution, lignocellulose degradation, secondary metabolites biosynthesis, and plant-pathogenic (Chen et al., 2012; Kües et al., 2015; Zhu et al., 2015; Dhillon et al., 2021; Jiang et al., 2021; Lin et al., 2021; Liu et al., 2021; Sun et al., 2022a).

In view of the demand for health preservation and the utilization of biological resources, the mycologists were devoted to explore the potential species resources of Ganodermataceae. Since the first introduction of Ganodermataceae, the taxonomy and phylogeny studies of this family have been conducted over the past 100 years, and now the number of genera has increased from 2 to 14 (Murrill, 1905; Donk, 1948; Imazeki, 1952; Steyaert, 1972; Costa-Rezende et al., 2017, 2020; Sun et al., 2020, 2022b). Besides, the rise of species diversity is impressive but uneven. *Ganoderma*, as the biggest genus in this family, has expanded to 188 species based on credible morphological and phylogenetic evidence; however, the sum of species number of the other 13 genera is only half of that of *Ganoderma* (Ryvarden, 2020; Wu et al., 2020; Decock and Ryvarden, 2021; He et al., 2022; Sun et al., 2022b; Vinjusha and Kumar, 2022).

Sun et al. (2020) clarified the taxonomy and phylogeny of *Amauroderma* s. lat. in Ganodermataceae, in which *Sanguinoderma* was established with *S. rude* as type species, and five new species were presented based on the morphological and multi-gene phylogenetic evidence. The distinguished characters of *Sanguinoderma* are the dull pileal surface, the color of fresh pore surface changing to blood red when bruised, and the double-walled basidiospores with obvious spinules on endospore walls (Sun et al., 2020). The phylogenetic tree showed that *Sanguinoderma rugosum* was performed as two lineages with high support; yet, no morphological differences between them were observed. Sun et al. (2022b) evaluated 22 specimens with color-changed pore surfaces and described six new species of *Sanguinoderma*. Unfortunately, the differentiation in *S. rugosum* was ignored again due to the inappreciable differences. In fact, the variable morphological description of *S. rugosum* from different collections was proposed 40 years ago, for example, thin to thick and flexible to rigid pilei, dark brown to fuscous brown or black pileal surface with or without concentric zones in variable color, globose to subglobose basidiospores from 6.5 to 13 μm × 7 to 11 μm and so on Ryvarden and Johansen (1980), Corner (1983), Núñez and Ryvarden (2000). These differences indicated that the *S. rugosum* complex should be further excavated to solve the problem of subspecies differentiation.

During our investigations of *Sanguinoderma*, numerous specimens of *S. rugosum* complex were collected. The macro-/micro-morphological differences and phylogenetic relationships reflected their divergences indeed. Based on the morphological and phylogenetic analyses, five species were discovered in the *S. rugosum* complex, *Sanguinoderma leucomarginatum* was described as a new species, and another three species were identified as suspected new species due to their sterile basidiomata. In addition, *Amauroderma preussii* was transferred to *Sanguinoderma* as a new combination.

## Materials and methods

### Morphological study

The studied specimens are deposited at the herbaria of the Institute of Microbiology, Beijing Forestry University (BJFC, Beijing, China), and the Institute of Microbiology, Chinese Academy of Sciences, China (HMAS). Macro-morphological descriptions of the taxa were based on field notes and herbarium specimens. Micro-morphological data were obtained from dried specimens and observed under a compound microscope following by Sun et al. (2022b) and Liu et al. (2022). Sections were studied at a magnification up to 1,000× using a Nikon Digital Sight DS-Fi2 microscope (Nikon Corporation, Tokyo, Japan) and quantified by the Image-Pro Plus 6.0 software (Media Cybernetics, Silver Spring, USA). Special color terms followed Petersen (1996). Morphological descriptions and abbreviations used in this study followed Cui et al. (2019) and Sun et al. (2022b).

### DNA extraction, amplification, and sequencing

The total genomic DNA was extracted from the dried specimens using CTAB rapid plant genome extraction kit-DN14 (Aidlab Biotechnologies Co., Ltd, Beijing, China) and a FH plant DNA kit II (Demeter Biotech Co., Ltd., Beijing, China). The detailed methods of DNA extraction and polymerase chain reaction (PCR) were according to the manufacturer's instructions with some modifications (Sun et al., 2020; Liu et al., 2022). The internal transcribed spacer regions (ITS) were amplified with primer pairs ITS5 and ITS4 (White et al., 1990). The large subunit of nuclear ribosomal RNA gene (nLSU) was amplified with primer pairs LR0R and LR7, and the primer LR5 was used sometimes as an alternative to LR7 (Vilgalys and Hester, 1990). The second subunit of RNA polymerase II (*rpb2*) was amplified with primer pairs fRPB2-5F and fRPB2-7CR (Liu et al., 1999). The translation elongation factor 1-α gene (*tef1*) was amplified with primer pairs EF1-983F and EF1-1567R (Rehner and Buckley, 2005). The small subunit mitochondrial rRNA gene (mtSSU) was amplified with primer pairs MS1 and MS2 (White et al., 1990). The small subunit nuclear ribosomal RNA gene (nSSU) was amplified with primer pairs PNS1 and NS41 (White et al., 1990).

The PCR volume contained 1 μl each primer, 1 μl extracted DNA, 12 μl ddH_2_O, and 15 μl 2 × EasyTaq PCR SuperMix (TransGen Biotech Co., Ltd., Beijing, China). The PCR cycling schedules for six-gene regions of ITS, nLsu, *rpb2, tef1*, nSSU, and mtSSU was followed by Sun et al. (2020, 2022b). The PCRs were performed on S1000™ Thermal Cycler (Bio-Rad Laboratories, California, USA), and the PCR products were purified and sequenced with the same primers at the Beijing Genomics Institute (BGI), China. All sequences used in this study were deposited at GenBank and are listed in Table 1.

**Table 1:** Taxa information and GenBank accession numbers of the sequences used in this study.

**Species**	**Voucher**	**Locality**	**GenBank accession no**.						**References**
			**ITS**	**LSU**	***rpb2***	***tef1***	**mtSSU**	**nSSU**	
*Sanguinoderma bataaense*	Dai 10746	Hainan	MK119832	MK119911	MK121511	MK121581	MZ352801	MZ355267	Sun et al., 2020, 2022b
	Cui 6285	Hainan	MK119831	MK119910	MK121537	MK121580	MZ352793	MZ355238	Sun et al., 2020, 2022b
	Dai 7862	Hainan	KJ531658	–	–	–	–	–	Li and Yuan, 2015
*S. elmerianum*	HMAS 133187	Yunnan	MK119834	MK119913	–	–	MZ352824	MZ355234	Sun et al., 2020, 2022b
	Dai 20634	Yunnan	MZ354875	MZ355082	–	MZ221724	MZ352821	MZ355148	Sun et al., 2022b
	Cui 8940	Guangdong	MK119833	MK119912	–	–	MZ352812	MZ355305	Sun et al., 2020, 2022b
*S. flavovirens*	Cui 16935^**T**^	Zambia	–	MK119914	MK121532	MK121582	MZ352811	MZ355254	Sun et al., 2020, 2022b
*S. guangdongense*	Cui 17259^**T**^	Guangdong	MZ354877	MZ355123	MZ358834	MZ221726	MZ352816	MZ355139	Sun et al., 2022b
	Dai 16724	Thailand	MZ354876	MZ355117	MZ358833	MZ221725	MZ352815	MZ355271	Sun et al., 2022b
	Dai 20419	Yunnan	MZ354890	MZ355083	MZ358835	MZ221727	MZ352818	MZ355155	Sun et al., 2022b
*S. infundibulare*	Dai 18149^**T**^	Guangdong	MK119847	MK119926	MK121529	MK121597	MZ352790	MZ355239	Sun et al., 2020, 2022b
	URM 450213	Ecuador	MK119849	MK119927	–	–	MZ352792	MZ355252	Sun et al., 2020, 2022b
	Cui 17238	Guangdong	OM780277	–	MZ358837	MZ221729	MZ352800	MZ355149	Sun et al., 2022b
*S. laceratum*	A5	India	MG383652	–	–	–	–	–	Unpublished
	Cui 8155^**T**^	Yunnan	MK119851	MK119928	–	–	MZ352810	–	Sun et al., 2020, 2022b
***S. leucomarginatum***	**Dai 12264**	**Yunnan**	**OP700311**	**OP700344**	**OP696845**	**OP696857**	**OP703259**	**OP700325**	**This study**
	**Dai 12377** ^**T**^	**Yunnan**	**OP700312**	**OP700345**	**OP696846**	**OP696860**	**OP703260**	**OP700326**	**This study**
	**Dai 12362**	**Yunnan**	**KU219986**	**KU220009**	**OP696847**	**OP696858**	**OP703261**	**OP700327**	**Song et al.**, **2016**
*S. longistipitum*	Dai 20696^**T**^	Yunnan	MZ354881	MZ355084	–	MZ221732	MZ352822	MZ355145	Sun et al., 2022b
	Cui 13903	Hainan	MZ354882	MZ355114	MZ358839	MZ221733	MZ352809	MZ355301	Sun et al., 2022b
	Dai 16635	Thailand	MZ354883	MZ355120	MZ358840	MZ221734	MZ352802	MZ355260	Sun et al., 2022b
*S. melanocarpum*	Dai 18512	Malaysia	MZ354888	MZ355118	–	MZ221735	MZ352794	MZ355313	Sun et al., 2022b
	Dai 18603^**T**^	Malaysia	MZ354889	MZ355113	MZ358841	MZ221736	MZ352796	MZ355281	Sun et al., 2022b
*S. microporum*	Cui 13851^**T**^	Hainan	MK119854	MK119933	MK121512	MK121602	MZ352797	MZ355270	Sun et al., 2020, 2022b
	Cui 14022	Guangxi	MK119856	MK119935	MK121515	MK121604	MZ352798	MZ355298	Sun et al., 2020, 2022b
	Cui 16335	Guangxi	MK119857	MK119936	MK121514	MK121605	OP703262	OP700328	Sun et al., 2020; this study
	Cui 14001	Guangxi	MK119855	MK119934	MK121513	MK121603	OP703263	OP700329	Sun et al., 2020; this study
*S. microsporum*	Dai 16726^**T**^	Thailand	–	MZ355119	–	MZ221737	MZ352795	MZ355272	Sun et al., 2022b
	Cui 13897	Hainan	MZ354878	MZ355127	–	MZ221739	MZ352804	MZ355300	Sun et al., 2022b
	Cui 13901	Hainan	MZ354879	MZ355121	–	MZ221738	MZ352803	MZ355299	Sun et al., 2022b
*S. perplexum*	Cui 6496	Hainan	KJ531650	KU220001	MK121538	MK121583	MZ352825	MZ355263	Li and Yuan, 2015; Sun et al., 2022b
	Cui 6554	Hainan	MK119835	MK119915	MK121540	MK121585	MZ352826	MZ355264	Sun et al., 2020, 2022b
	Dai 10811	Hainan	KJ531651	KU220002	MK121539	MK121584	MZ352827	MZ355302	Li and Yuan, 2015; Sun et al., 2022b
	Wei 5562	Hainan	KJ531652	–	–	–	–	–	Li and Yuan, 2015
***S. preussii***	**HMAS 130806**	**Yunnan**	**OP700313**	**OP700346**	**–**	**–**	**OP703264**	**OP700330**	**This study**
	**Dai 20438**	**Yunnan**	**OP700314**	**OP700347**	**OP696848**	**OP696869**	**OP703265**	**OP700331**	**This study**
	**Dai 20622**	**Yunnan**	**OP700315**	**OP700348**	**–**	**OP696862**	**OP703266**	**OP700332**	**This study**
	**Dai 20624**	**Yunnan**	**OP700316**	**OP700349**	**–**	**OP696863**	**OP703267**	**OP700333**	**This study**
*S. reniforme*	Cui 16511^**T**^	Zambia	MK119850	MK119929	MK121531	MK121599	–	MZ355322	Sun et al., 2020, 2022b
*S. rude*	MEL 2317411	Australia	MK119842		MK121524	MK121592	MZ352819	MZ355306	Sun et al., 2020, 2022b
	DHCR457	Brazil	MN077517	MN077551	–	MN061693	–	–	Costa-Rezende et al., 2020
	Cui 16592	Australia	MK119836	MK119916	MK121521	MK121586	MZ352924	MZ355307	Sun et al., 2020, 2022b
*S. rugosum*	Cui 16160	Guangxi	MK119845	MK119924	MK121520	MK121595	OP703268	OP700334	Sun et al., 2020; this study
	Cui 16337	Guangxi	MK119844	MK119923	MK121519	MK121594	OP703269	OP700335	Sun et al., 2020; this study
	Cui 17260	Guangdong	OP700317	OP700350	OP696849	OP696859	OP703270	OP700336	This study
	Cui 14033	Guangxi	OP700318	OP700351	OP696850	OP696864	OP703271	OP700337	This study
	Cui 8972	Guangdong	OP700319	OP700352	OP696852	OP696861	OP703272	OP700338	This study
	Dai 16437	Hainan	OP700320	OP700353	OP696853	OP696866	OP703273	OP700339	This study
	Cui 6185	Hainan	–	OP700354	OP696851	OP696867	OP703274	OP700340	This study
*S. sinuosum*	MEL 2366586^**T**^	Australia	MK119852	MK119930	MK121527	MK121600	MZ352920	MZ355261	Sun et al., 2020, 2022b
	MEL 2341763	Australia	MK119853	MK119931	MK121525	MK121601	MZ352820	MZ355291	Sun et al., 2020, 2022b
*Sanguinoderma* sp.1	Cui 11017	Yunnan	OP700321	OP700355	OP696854	OP696865	OP703275	OP700341	This study
*Sanguinoderma* sp.1	HMAS 59720	Guizhou	OP700322	OP700356	–	OP696870	OP703276	OP700342	This study
*Sanguinoderma* sp.2	Cui 8795	Guangdong	MK119843	MK119922	MK121516	MK121516	MZ352799	MZ355266	Sun et al., 2020, 2022b
*Sanguinoderma* sp.2	Dai 20582	Yunnan	MZ354887	MZ355085	MZ358842	MZ221741	MZ352823	MZ355156	Sun et al., 2022b
*Sanguinoderma* sp.2	Cui 9011	Guangdong	KJ531664	KU220010	MK121517	KU572504	MZ352805	MZ355237	Li and Yuan, 2015; Sun et al., 2022b
*Sanguinoderma* sp.2	Cui 9012	Guangdong	KJ531665	KU220011	MK121518	KU572503	MZ352807	MZ355269	Li and Yuan, 2015; Sun et al., 2022b
*Sanguinoderma* sp.2	Cui 9066	Guangdong	MZ354884	MZ355122		MZ221740	MZ352806	MZ355268	Sun et al., 2022b
*Sanguinoderma* sp.3	Dai 16810	Thailand	OP700323	OP700357	OP696855	OP696868	OP703277	OP700343	This study
*Sanguinoderma* sp.3	Cui 18251	Malaysia	OP700324	OP700358	OP696856	OP696871	OP703278	–	This study
*S. tricolor*	Cui 18242	Malaysia	MZ354992	MZ355099	MZ358843	MZ221743	MZ352829	MZ355303	Sun et al., 2022b
	Cui 18292^**T**^	Malaysia	–	MZ355101	–	MZ221742	MZ352828	MZ355273	Sun et al., 2022b
	Dai 18574	Malaysia	MZ354993	MZ355102	MZ358844	MZ221744	MZ352830	MZ355265	Sun et al., 2022b
*Magoderna subresinosum*	Dai 18626	Malaysia	MK119823	MK119902	MK121507	MK121571	MZ352831	MZ355211	Sun et al., 2020, 2022b
	Cui 18262	Malaysia	MZ354871	MZ355088	–	–	MZ352832	MZ355258	Sun et al., 2022b

Species in bold are new species or new combinations.

### Phylogenetic analyses

The ITS, nLSU, *rpb2, tef1*, mtSSU, and nSSU sequences used in this study were combined into a dataset. *Magoderna subresinosum* was used as the outgroup, which is a sister clade with *Sanguinoderma* (Sun et al., 2022b). Phylogenetic analyses used in this study followed the approach of Cui et al. (2019). These sequences were aligned in online MAFFT v. 7 (Katoh et al., 2019; https://mafft.cbrc.jp/alignment/server/) and manually adjusted using BioEdit (Hall, 1999). Each alignment of ITS, nLSU, *rpb2, tef1*, mtSSU, and nSSU was catenated in Mesquite (Maddison and Maddison, 2017). The congruencies of six-gene loci were evaluated with the partition homogeneity test (PHT) (Farris et al., 1994) using PAUP v. 4.0b10 (Swofford, 2002) under 1,000 homogeneity replicates. The best-fit evolutionary model was calculated in MrModeltest v. 2.3 (Nylander, 2008) using hierarchical-likelihood ratio tests (hLRTs) and Akaike information criterion (AIC) strategies.

Based on the combined dataset, the maximum-likelihood (ML) analyses were conducted in RAxML-HPC v. 8.2.3 (Stamatakis, 2014). The best topology was obtained during 1 000 ML searches under the GTRGAMMA model, and 1,000 rapid bootstrap replicates were run with the GTRCAT model to assess the ML bootstrap values of the nodes. Bayesian inference analyses were calculated using MrBayes v. 3.1.2 (Ronquist and Huelsenbeck, 2003). The analyses were run with four Markov chains, starting trees for 12 M generations until the average standard deviation of split deviation frequency < 0.01, and sampled every 100 generations. The first 25% of the sampled trees were discarded as burn-in, and the remaining ones were used to reconstruct a majority rule consensus and calculate Bayesian posterior probability (BPP) of the clades.

All trees were visualized in FigTree v. 1.4.2 (http://tree.bio.ed.ac.uk/software/figtree/). The branches received ML bootstrap ≥ 70%, and Bayesian posterior probabilities ≥0.95 were regarded as credibly supported. The final alignments and the phylogenetic tree were deposited in TreeBASE (http://www.treebase.org), under accession ID: 29788 (http://purl.org/phylo/treebase/phylows/study/TB2:S29788).

## Results

### Molecular phylogeny

In this study, 340 sequences of ITS, nLSU, *rpb2, tef1*, mtSSU, and nSSU were used to construct phylogenetic trees of *Sanguinoderma*, including 61 ITS sequences, 60 nLSU sequences, 44 *rpb2* sequences, 56 *tef1* sequences, 60 mtSSU sequences, and 59 nSSU sequences. The inferred sequences were obtained from 65 specimens representing 21 taxa in *Sanguinoderma* and *Magoderna subresinosum* as the outgroup. The combined six-gene (ITS+nLSU+*rpb2*+*tef1*+mtSSU+nSSU) sequence datasets had an aligned length of 5 017 total characters including gaps, of which 4 374 are constant, 207 are variable and parsimony-uninformative, and 436 are parsimony-informative.

The partition homogeneity test indicated all six different genes displayed a congruent phylogenetic signal (*P* = 1.00). The best-fit evolutionary models selected by MrModeltest v. 2.3 for each region of the six genes were K80+I (ITS1), K80 (5.8S), HKY+G (ITS2), GTR+I (nLSU), K80 (*rpb2* introns), K80+I (*rpb2* 1st codon), GTR+I+G (*rpb2* 2nd codon), K80+G (*tef1* introns), HKY+I (*tef1* 1st codon), SYM+I+G (*tef1* 2nd codon), GTR+G (*tef1* 3rd codon), HKY+I+G (mtSSU), and GTR (nSSU). These models were applied in Bayesian analyses for the combined dataset.

**Figure 1:**
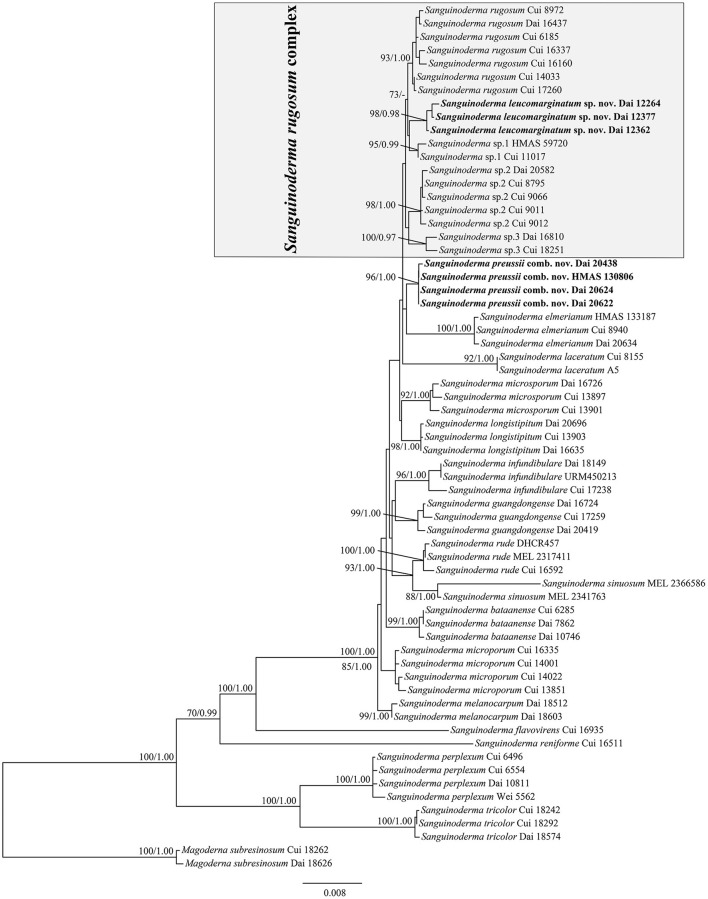
Maximum-likelihood (ML) analyses of *Sanguinoderma* based on the dataset of ITS+nLSU+*rpb2*+*tef1*+mtSSU+nSSU. Branches are labeled with maximum-likelihood bootstrap values equal to or higher than 70% and Bayesian posterior probability values equal to or higher than 0.95. New species or combinations are in bold.

The average standard deviation of split frequencies in the Bayesian analyses reached 0.004273. The ML analyses resulted in a similar topology as Bayesian analyses, and only the ML topology with the calculated values is shown in Figure 1. The lineages presented in the phylogenetic tree were *S. leucomarginatum* as new species (98% ML, 0.98 BPP), *S. preussii* as new combination (96% ML, 1.00 BPP), *S. bataanense* (99% ML, 1.00 BPP), *S. elmerianum* (100% ML, 1.00 BPP), *S. flavovirens, S. guangdongense* (99% ML, 1.00 BPP), *S. laceratum* (92% ML, 1.00 BPP), *S. longistipitum* (98% ML, 1.00 BPP), *S. infundibulare* (96% ML, 1.00 BPP), *S. melanocarpum* (99% ML, 1.00 BPP), *S. microporum* (88% ML, 1.00 BPP), *S. microsporum* (92% ML, 1.00 BPP), *S. perplexum* (100% ML, 1.00 BPP), *S. reniforme, S. rude* (100% ML, 1.00 BPP), *S. rugosum* (93% ML, 1.00 BPP), *S. sinuosum* (88% ML, 1.00 BPP), *S. tricolor* (100% ML, 1.00 BPP), and three undetermined taxa: *Sanguinoderma* sp.1 (95% ML, 0.99 BPP), *Sanguinoderma* sp.2 (98% ML, 1.00 BPP), and *Sanguinoderma* sp.3 (100% ML, 0.97 BPP). *Sanguinoderma rugosum* complex comprised of *S. rugosum, S. leucomarginatum, Sanguinoderma* sp.1, *Sanguinoderma* sp.2, and *Sanguinoderma* sp.3, sharing the similar morphological characters.

### Taxonomy

**Figure 2:**
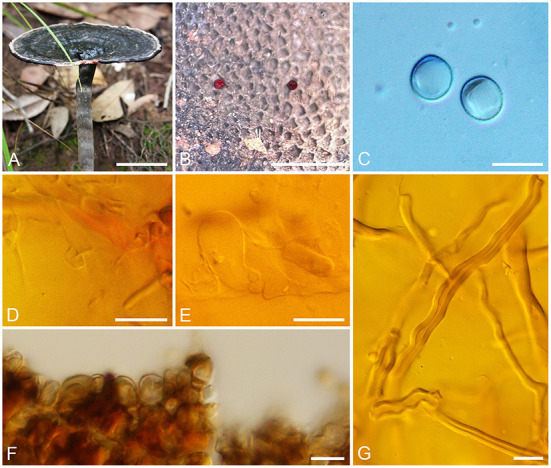
Basidiomata and microscopic structures of *Sanguinoderma leucomarginatum*. **(A)** Basidiomata. **(B)** Pores. **(C)** Basidiospores. **(D)** Clamp connections on generative hyphae. **(E)** Basidioles. **(F)** Pileipellis. **(G)** Skeletal hyphae. Scale bars: **(A)** = 2 cm, **(B)** = 1 mm, **(C–G)** = 10 μm.

***Sanguinoderma leucomarginatum*** B. K. Cui and Y. F. Sun, sp. nov. (Figure 2)

MycoBank number: MB 846192

*Diagnosis*: Differs from other species in the genus by having near orbicular pilei with white to buff margin when fresh and clavate apical cells of pileipellis with septa.

*Etymology*: *leucomarginatum* (Lat.) refers to the white to buff margin of pilei.

*Holotype*: CHINA. Yunnan Province, Pu'er City, Laiyanghe Nature Reserve, on ground of forest, 9 June 2011, Yu-Cheng Dai, Dai 12377 (BJFC 010657).

*Description*: Basidiomata annual, laterally stipitate, hard corky to woody hard. Pilei solitary, near orbicular, up to 8 cm in diameter and 7-mm thick. Pileal surface fawn to vinaceous gray or near black, margin white to buff, dull, glabrous, with fuscous concentric zones or edges, and radial wrinkles near the margin; margin acute to obtuse, entire, slightly incurved and wavy when dry. Pore surface becoming blood red when bruised and then quickly darkening, pale mouse gray to ash-gray when dry; pores circular to angular, 5–6 per mm; dissepiments slightly thick, entire. Context cream to buff yellow, with dark melanoid lines, hard corky, up to 3-mm thick. Tubes light vinaceous gray to ash-gray, up to 3-mm long. Stipe clay buff to fawn, cylindrical and hollow, up to 8.5-cm long and 8 mm in diameter.

Hyphal system trimitic; generative hyphae with clamp connections, all hyphae IKI–, CB+; tissues darkening in KOH. Generative hyphae in context colorless, thin-walled, 3–6 μm in diameter; skeletal hyphae in context faint yellow, thick-walled with a wide to narrow lumen or sub-solid, arboriform and flexuous, 3–7 μm in diameter; binding hyphae in context faint yellow, sub-solid, branched and flexuous, up to 2 μm in diameter. Generative hyphae in tubes colorless, thin-walled, 3–6 μm in diameter; skeletal hyphae in tubes faint yellow, thick-walled with a wide to narrow lumen or sub-solid, arboriform and flexuous, 3–6 μm in diameter; binding hyphae in tubes faint yellow, sub-solid, branched, and flexuous, up to 2 μm in diameter. Pileipellis composed of clamped generative hyphae, thick-walled, apical cells clavate with septa, slightly inflated, yellow to reddish brown, about 40–70 × 4–7 μm, forming a regular palisade. Cystidia and cystidioles absent. Basidia barrel-shaped, colorless, thin-walled, 14–20 × 14–16 μm; basidioles in shape like the basidia, colorless, thin-walled, 12–23 × 6–15 μm. Basidiospores subglobose to broadly ellipsoid, pale yellow, IKI–, CB+, double-walled with slightly thick walls, exospore wall smooth, endospore wall with dense spinules (8.5–)8.8–10.1 × (7.4–)7.8–9 μm, L = 9.32 μm, W = 8.3 μm, Q = 1.12 (n = 60/1).

*Additional specimens examined*: CHINA. Yunnan Province, Pu'er City, Laiyanghe Nature Reserve, on ground of angiosperm forest, 9 June 2011, Yu-Cheng Dai, Dai 12264 (BJFC 010547), Dai 12390 (BJFC 010670); on root of Castanea, 9 June 2011, Yu-Cheng Dai, Dai 12362 (BJFC 010642); Jinghong City, Xishuangbanna Nature Reserve, on ground of forest, 7 June 2011, Yu-Cheng Dai, Dai 12324 (BJFC 010605).

*Notes*: *Sanguinoderma leucomarginatum* was described from Yunnan Province of Southwestern China. It is distinguished by its more or less orbicular pilei with white to buff margin when fresh and the clavate apical cells of pileipellis with septa. According to the previous studies, four species of *Sanguinoderma* had been reported from Yunnan Province, viz. *S. elmerianum, S. guangdongense, S. laceratum*, and *S. longistipitum* (Sun et al., 2020, 2022b). Compared to these species, *S. leucomarginatum* has the medially sized pores (5–6 per mm) with entire dissepiments, the stipe in medium length (up to 8.5 cm), and smaller basidiospores (8.8–10.1 × 7.8–9 μm). In the phylogenetic tree, *S. leucomarginatum* was presented as a distinct lineage with high support (Figure 1).

**Figure 3:**
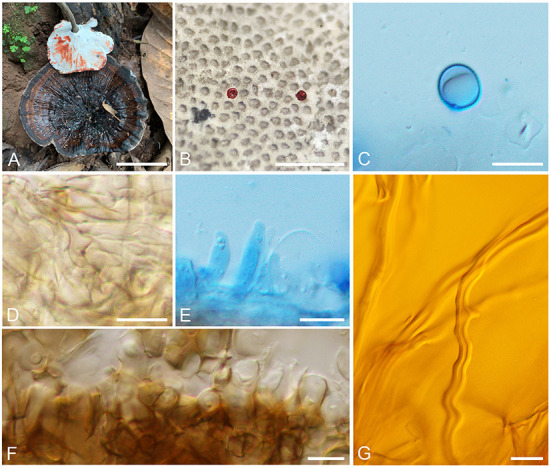
Basidiomata and microscopic structures of *Sanguinoderma preussii*. **(A)** Basidiomata. **(B)** Pores. **(C)** Basidiospores. **(D)** Clamp connections on generative hyphae. **(E)** Cystidioles. **(F)** Pileipellis. **(G)** Skeletal hyphae. Scale bars: **(A)** = 3 cm, **(B)** = 1 mm, **(C–G)** = 10 μm.

***Sanguinoderma preussii*** (Henn.) B. K. Cui and Y. F. Sun, comb. nov. (Figure 3)

MycoBank number: MB 846193

*Basionym*: *Ganoderma preussii* Henn., Bot. Jb. 14(4): 342 (1891).

=*Amauroderma preussii* (Henn.) Steyaert, Persoonia 7(1): 107 (1972).

=*Fomes preussii* (Henn.) Sacc., Syll. fung. (Abellini) 11: 89 (1895).

=*Scindalma preussii* (Henn.) Kuntze, Revis. gen. pl. (Leipzig) 3(3): 519 (1898).

=*Polyporus preussii* (Henn.) Lloyd, Mycol. Writ. 3 (Syn. Stip. Polyporoids) (Cincinnati): 124 (1912).

=*Ganoderma rubeolum* Bres., Mycologia 17(2): 73 (1925).

=*Ganoderma sikorae* Bres., Annln K. K. naturh. Hofmus. Wien 26: 157 (1912).

=*Polyporus salebrosus* Lloyd, Mycol. Writ. (Cincinnati) 4(Letter 42): 14 (1912).

=*Polyporus zambesianus* Lloyd, Mycol. Writ. 3 (Syn. Stip. Polyporoids) (Cincinnati): 128 (1912).

=*Polyporus rugosissimus* Lloyd, Mycol. Writ. (Cincinnati) 4(Letter 48): 3 (1913).

=*Ganoderma puberulum* Pat., Bull. Soc. mycol. Fr. 30(3): 343 (1914).

=*Fomes versicolor* Bres., in Beeli, Bull. Jard. bot. État Brux. 8: 91 (1922).

*Description*: Basidiomata annual, centrally stipitate, hard corky to woody hard. Pilei solitary, funnel-shaped, up to 10.5 cm in diameter and 3-mm thick. Pileal surface grayish brown, dull, glabrous, with black and concentric zones and radial wrinkles; margin acute, entire, petaloid, strongly incurved, and wavy when dry. Pore surface becoming to blood red when bruised and then quickly darkening, white to cream when dry; pores circular to angular or irregular, 6–7 per mm; dissepiments medially thick, entire. Context buff yellow, with dark melanoid lines, hard corky, up to 1-mm thick. Tubes ash-gray, up to 2-mm long. Stipe grayish brown, cylindrical, and hollow, up to 11.5-cm long and 8 mm in diameter.

Hyphal system trimitic; generative hyphae with clamp connections, all hyphae IKI–, CB+; tissues are darkening in KOH. Generative hyphae in context colorless, thin-walled, 3–4 μm in diameter; skeletal hyphae in context pale yellow, thick-walled with a wide to narrow lumen or sub-solid, arboriform and flexuous, 3–7 μm in diameter; binding hyphae in context pale yellow, sub-solid, branched, and flexuous, up to 2 μm in diameter. Generative hyphae in tubes colorless, thin-walled, 4–5 μm in diameter; skeletal hyphae in tubes pale yellow, thick-walled with a wide to narrow lumen or sub-solid, arboriform and flexuous, 4–6 μm in diameter; binding hyphae in tubes pale yellow, sub-solid, branched and flexuous, up to 2 μm in diameter. Pileipellis composed of clamped generative hyphae, thick-walled to sub-solid, apical cells clavate, inflated, pale yellow to yellowish brown, about 45–65 × 5–8 μm, forming a regular palisade. Cystidia absent; cystidioles clavate and apices constricted, colorless, thin-walled, 12–24 × 2–4 μm. Basidia near orbicular to barrel-shaped, colorless, thin-walled, 15–23 × 11–12 μm; basidioles barrel-shaped to clavate, colorless, thin-walled, 16–22 × 7–15 μm. Basidiospores subglobose to broadly ellipsoid, pale yellow, IKI–, CB+, double-walled with slightly thick walls, exospore wall smooth, endospore wall with dense spinules, 9–10.5(−10.8) × 8–9(−9.5) μm, L = 9.54 μm, W = 8.46 μm, Q = 1.13 (n = 60/2).

*Specimens examined*: THAILAND. Chiang Rai, Mae Salong Nok, on ground of angiosperm forest, 22 July 2016, Yu-Cheng Dai, Dai 16646 (BJFC 022756); on ground of forest, 24 July 2016, Yu-Cheng Dai, Dai 16725 (BJFC 022832). CHINA. Yunnan Province, Pu'er City, Pu'er Forestry Park, on ground of forest, 17 August 2019, Yu-Cheng Dai, Dai 20438 (BJFC 032106), Dai 20456 (BJFC 032124), Dai 20467 (BJFC 032135), Dai 20468 (BJFC 032136); Mengla County, Shangyong Nature Reserve, on ground of forest, 20 August 2019, Yu-Cheng Dai, Dai 20622 (BJFC 032289), Dai 20624 (BJFC 032291); Bakaxiaozhai Nature Reserve, on ground, 5 August 2003, Tie-Zheng Wei, HMAS 130806.

*Notes*: *Ganoderma preussii* was described from Cameroon and temporarily transferred to *Amauroderma* in Steyaert (1972) by its dull pileal surface and double-walled basidiospores without truncated apex. Here, *A. preussii* was transferred to *Sanguinoderma* due to the color-changed pore surface when bruised. The specimens used in this study were collected from East Asia, and the morphological characters of basidiomata are mostly consistent with the original description of *A. preussii* (Steyaert, 1972). However, Steyaert (1972) mentioned that the hyphae of pileipellis extend externally free and anticlinal at the base, while the structural characters of pileipellis of specimens observed in this study are forming as a palisade, which are similar to most species in *Amauroderma* s. lat.

*Sanguinoderma infundibulare* is another species with funnel-shaped pilei in *Sanguinoderma*, and it can be characterized by the yellowish brown and tomentose pileal surface with uncurved margin and large basidiospores (10.2–12 × 9–10.2 μm; Sun et al., 2022b). Besides, *S. preussii* and *S. infundibulare* were supported as two distinct lineages in the phylogenetic tree (Figure 1).

## Discussion

In this study, the multi-gene phylogenetic analyses of *Sanguinoderma* were conducted based on the combined dataset of ITS+nLSU+*rpb2*+*tef1*+mtSSU+nSSU sequences. In the phylogenetic tree, 21 taxa of *Sanguinoderma* clustered together with high support (100% ML, 1.00 BPP; Figure 1), in which 16 species were shown as well-supported respective lineages in accordance with previous studies by Sun et al. (2020, 2022b).

Sun et al. (2020, 2022b) have improved the classification of *Sanguinoderma* and reported 16 species in the genus with detailed morphological and phylogenetic evidence, while the differentiation in phylogeny of *Sanguinoderma rugosum* was still not studied. The variable morphological characters observed from different collections (Ryvarden and Johansen, 1980; Corner, 1983; Núñez and Ryvarden, 2000) provided an auxiliary basis for this divergence. During this study, more than 80 specimens were collected from East Asia, which were identified as *S. rugosum* for the first time. These specimens can be divided into five groups roughly in the analysis tests, and more concise lineages were presented in this article with high support (Figure 1). We treated the five lineages as five different taxa of the *S. rugosum* complex, which are similar in morphology.

*Sanguinoderma rugosum* as the core species of this complex is easily confused in morphology with the other four taxa, except the deeply concentric furrows on pileal surface, clavate cystidioles, and lager basidiospores (9.5–11.6 × 8–9.5 μm). *Sanguinoderma leucomarginatum* was separated from other taxa of the *S. rugosum* complex according to its white to buff pileal margin with fuscous concentric zones or edges, cream to buff context, absent cystidioles, and smaller basidiospores (8.8–10.1 × 7.8–9 μm). The other morphological characters, such as the wrinkled pileal surface, pale mouse gray to ash-gray pore surface when dry, and 5–6 pores per mm, are indistinguishable from the other four taxa. The other three suspected new species were discovered in this study based on the morphological differences and independent phylogenetic relationships. However, the failure to observe the mature basidiospores in morphological studies was the biggest obstacle to clarify the taxonomic status of these species; these three suspected new species were treated as undescribed taxa due to the sterile specimens, even though the structure of pileipellis in *Sanguinoderma* sp.1, the thickness of pore dissepiments in *Sanguinoderma* sp.2, and the color of pore surface in *Sanguinoderma* sp.3 can distinguish them availably (Table 2). The problem of the sterility of specimens is still unavoidable in taxonomic studies.

**Table 2:** Main morphological characters of species in *Sanguinoderma rugosum* complex.

**Species**	**Pilei**	**Pore surface (when dry)**	**Pore dissepiments**	**Pileipellis**	**Cystidioles**	**Basidiospores**
*S. leucomarginatum*	Fuscous concentric zones or edges and radial wrinkles near the cream margin	Pale mouse gray to ash-gray	Slightly thick	Apical cells clavate with septa, slightly inflated	Absent	8.8–10.1 × 7.8–9 μm
*S. rugosum*	Concentric furrows and radial wrinkles, navel-shaped center	White to cream or buff	Slightly thick	Apical cells clavate, inflated	Clavate and apexes constricted	9.5–11.6 × 8–9.5 μm
*Sanguinoderma* sp.1	Concentric zones and radial wrinkles	Pale mouse gray to ash-gray	Slightly thick	Apical cells gelatinized, irregular	Absent	–
*Sanguinoderma* sp.2	Concentric furrows and radial wrinkles, navel-shaped center	Pale grayish white	Distinctly thick	Apical cells clavate, inflated	Absent	–
*Sanguinoderma* sp.3	Concentric zones and radial wrinkles	White to cram	Distinctly thick	Apical cells clavate, constricted	Absent	–

*Sanguinoderma preussii* can be easily distinguished by the funnel-shaped and thin pilei with an incurved margin-like petals. Hapuarachchi et al. (2018) examined the specimens of *S. preussii* collected from Xiengkhouang Province in Laos and Hainan Province in China, but the recorded size of pores (2–4 per mm) is quite different from the observation in this study (6–7 per mm). The funnel-shaped pilei were also observed in *Amauroderma wuzhishanense* according to the description by Zhao and Zhang (1987), but the tubercles and broad radial wrinkles on pileal surface make *A. wuzhishanense* (= *A. rugosum*) different from the smooth pileal surface with lender radial wrinkles in *S. preussii*. The collections from East Asia enriched the distributions of *S. preussii*, and it implies that the species of *Sanguinoderma* may be widespread in Palaeotropics, such as *S. rugosum* and *S. rude*.

After the morphological and phylogenetic analyses, one new species called *S. leucomarginatum* was separated from *S. rugosum* complex. Besides, there are three suspected new species in *Sanguindoerma rugosum* complex without valid taxonomic status due to the sterile specimens. In addition, one new combination called *S. preussii* was transferred from *Amauroderma*. In summary, 18 species were accepted in *Sanguinoderma* around the world, in which 12 species were distributed in China; a key to accepted species of *Sanguinoderma* is provided. In further studies, more fertile specimens need to be collected to enrich the species diversity and clarify the taxonomic status of the suspected species.

## Key to accepted species of *Sanguinoderma*

(1) Pore dissepiments extremely thick………………………..2
(1) Pore dissepiments thin to distinctly thick…………………3
(2) Pileal surface pale yellowish brown, pore surface yellowish brown, context with dark melanoid lines……*S. microporum*
(2) Pileal surface rust brown to almost black, pore surface white to pale yellow, context without dark melanoid lines… …………………………………………………*S. tricolor*
(3) Pore dissepiments lacerate, tubes fascicular when dry…… ………………………………………………*S. laceratum*
(3) Pore dissepiments entire, tubes unchanged when dry…… …………………………………………………………4
(4) Pores less than or equal to 4 per mm……………………...5
(4) Pores more than 4 per mm………………………………7
(5) Pores sinuate; basidiospores more than 13.5 μm in length…………………………………………*S. sinuosum*
(5) Pores circular to irregular; basidiospores less than 13.5 μm in length…….……….……………………………………6
(6) Pore dissepiments thin; basidiospores globose to subglobose…………………………………..*S. bataanense*
(6) Pore dissepiments slightly thick; basidiospores subglobose to broadly ellipsoid……… …………………………..*S. rude*
(7) Basidiospores less than 6 μm in length…………… …………………………………………...*S. microsporum*
(7) Basidiospores more than 6 μm in length…………………8
(8) Pileal surface coal black; basidiospores slightly dextrinoid in Melzer's reagent…………………………*S. melanocarpum*
(8) Pileal surface brown to almost black; basidiospores IKI- in Melzer's reagent…………………………………………9
(9) Pilei funnel-shape………………………………………10
(9) Pilei flat…………….….….……………………………11
(10) Pileal margin uncurved; larger basidiospores (10.2–12 × 9–10.2 μm)…………………………………*S. infundibulare*
(10) Pileal margin strongly incurved; smaller basidiospores (9–10.5 × 8–9 μm)…………………………………*S. preussii*
(11) Basidiospores reniform…………….….………*S. reniforme*
(11) Basidiospores globose to subglobose or broadly ellipsoid…12
(12) Pore surface yellowish green when fresh………… ……………………………………………*S. flavovirens*
(12) Pore surface pale white to cream or pale grey……………13
(13) Cystidioles absent………………………………………14
(13) Cystidioles present……….….….………………………15
(14) Pileal margin white to buff; basidiospores less than 9 μm in width…………………………………*S. leucomarginatum*
(14) Pileal margin dark brown to nearly black; basidiospores more than 9 μm in width…………………………*S. elmerianum*
(15) Basidiomata sessile to subsessile; basidiospores more than or equal to 14 μm in length……………… ………………………………………………*S. perplexum*
(15) Basidiomata stipitate; basidiospores less than 14 μm in length………….….……………………………………16
(16) Pileal surface with shades of brown concentric zones and dense radial lines………………………...*S. guangdongense*
(16) Pileal surface with concentric furrows and radial wrinkles………………………………………………...17
(17) Basidiomata small, with lateral stipe; cystidioles fusiform…………………………………...*S. longistipitum*
(17) Basidiomata large, with central to lateral stipe; cystidioles clavate………………………….….….………*S. rugosum*.

## Data availability statement

The datasets presented in this study can be found in online repositories. The names of the repository/repositories and accession number(s) can be found in the article/supplementary material.

## Author contributions

B-KC designed the research. B-KC and Y-FS prepared the samples and drafted the manuscript. Y-FS and Y-XF conducted the molecular experiments and analyzed the data. All authors have read and agreed to the published version of the manuscript.
